# The impact of metagenomic analysis on the discovery of novel endolysins

**DOI:** 10.1007/s00253-025-13513-2

**Published:** 2025-05-24

**Authors:** Nikolaos Georgakis, Georgios E. Premetis, Panagiota Pantiora, Christina Varotsou, Charoutioun S. Bodourian, Nikolaos E. Labrou

**Affiliations:** Laboratory of Enzyme Technology, Department of Biotechnology, School of Applied Biology and Biotechnology, Agricultural University of Athens, 75 Iera Odos Street, Athens, 11855 Greece

**Keywords:** Biofilm, Metagenomics, Microbiome, Endolysins, Peptidoglycan hydrolases, Thermostability, Enzybiotics

## Abstract

**Abstract:**

Metagenomics has revolutionized enzyme discovery by enabling the study of genetic material directly from environmental samples, bypassing the need for microbial cultivation. This approach is particularly effective for identifying novel endolysins, phage-derived enzymes with antibacterial properties suited for therapeutic and industrial applications. Diverse ecosystems, such as biofilms, human microbiome, hot springs, and geothermal areas, serve as rich reservoirs for endolysins with traits like thermostability, broad-spectrum activity, specificity and resistance to harsh conditions. Functional metagenomics, complemented by bioinformatics, enables the discovery and annotation of previously uncharacterized endolysins. Examples of endolysins discovered from metagenomics analysis are discussed. Despite the challenges of analyzing complex microbial ecosystems and isolating target genes, metagenomics holds immense potential for uncovering innovative endolysins, paving the way for developing new biotechnological applications.

**Key points:**

*• Endolysins offer antibacterial potential for therapeutic and industrial use.*

*• Metagenomics enables discovery of novel endolysins from diverse ecosystems.*

*• Advances in tools and methods have accelerated novel endolysins discovery.*

## Introduction

The widespread and inappropriate use of antibiotics has led to the phenomenon of bacterial multidrug resistance, which poses a threat to the substantial progress made in healthcare (Dumont et al. 2024; Yuan et al. 2024). This urgent threat to human health necessitates the exploration of novel and innovative antibacterial strategies (Chang et al. 2022; Abbas et al. 2024; Derollez et al. 2024). Among the most promising strategies is the utilization of peptidoglycan hydrolases (PGHs), which specifically target and degrade the peptidoglycan (PG) layer of bacterial cell walls, ultimately resulting in bacterial lysis (Bhagwat et al. 2020; Khan et al 2024 Zhydzetski et al. 2024). PGHs were previously described as lysins, which are classified into endolysins, exolysins, and autolysins, based on their origin and function (Vázquez and Briers 2023; Vázquez et al. 2021). Endolysins are encoded by bacteriophages and hydrolyze the host bacterial cell wall at the initiation of the lytic cycle (Bhagwat et al. 2020). Exolysins refer to hydrolases secreted by bacteria to neutralize other bacterial species or strains within the same species (Khan et al. 2024a; Zhydzetski et al. 2024). Autolysins are hydrolases involved in the reorganization of the cell wall during growth and cell division (Brogan and Rudner 2023).

Endolysins have shown significant promise as antimicrobial agents due to their remarkable specificity, often targeting bacteria at the genus level and, in some cases, even at the strain level within the same species (Gutiérrez and Briers 2021; Oechslin et al. 2022; Vázquez et al. 2024). Several studies have investigated the effect of endolysins on both Gram-positive and Gram-negative bacteria (Gutiérrez and Briers 2021; Ho et al. 2022). However, due to differences in the structure of the cell wall between Gram-positive and Gram-negative bacteria, these enzymes appear to be more effective against Gram-positive bacteria, as they have direct access to the peptidoglycan layer (Carratalá et al. 2023; Sisson et al. 2024). However, some endolysins exhibit activity against both Gram-positive and Gram-negative bacteria (Jiang et al. 2021). When endolysins are produced by phages and act from within the host cell (i.e., after phage infection), the outer membrane does not pose a barrier—since the enzyme is already inside the cell envelope. In this case, the accessibility differences are less relevant. Instead, the structural differences in the peptidoglycan itself (e.g., composition, crosslinking, and thickness) and the mechanism of host lysis may play more important roles in determining endolysin effectiveness and specificity.

Although significant progress has been made in understanding the biochemistry of phage endolysins, their functional diversity and its implications for phage-host adaptation and evolutionary dynamics remain comparatively underexplored (Oechslin et al. 2022; Oechslin et al. 2024). Oechslin et al. (2022) demonstrated using CRISPR-Cas9 that the genetic exchange of endolysin genes between phages incurs minimal fitness costs within the same bacterial strain, but these costs increase when exchanged across different strains or species. Natural recombination and adaptive mutations can further enhance endolysin diversity and functionality, highlighting the evolutionary flexibility of endolysins and their potential for engineering as novel antimicrobial agents.

**Fig. 1:**
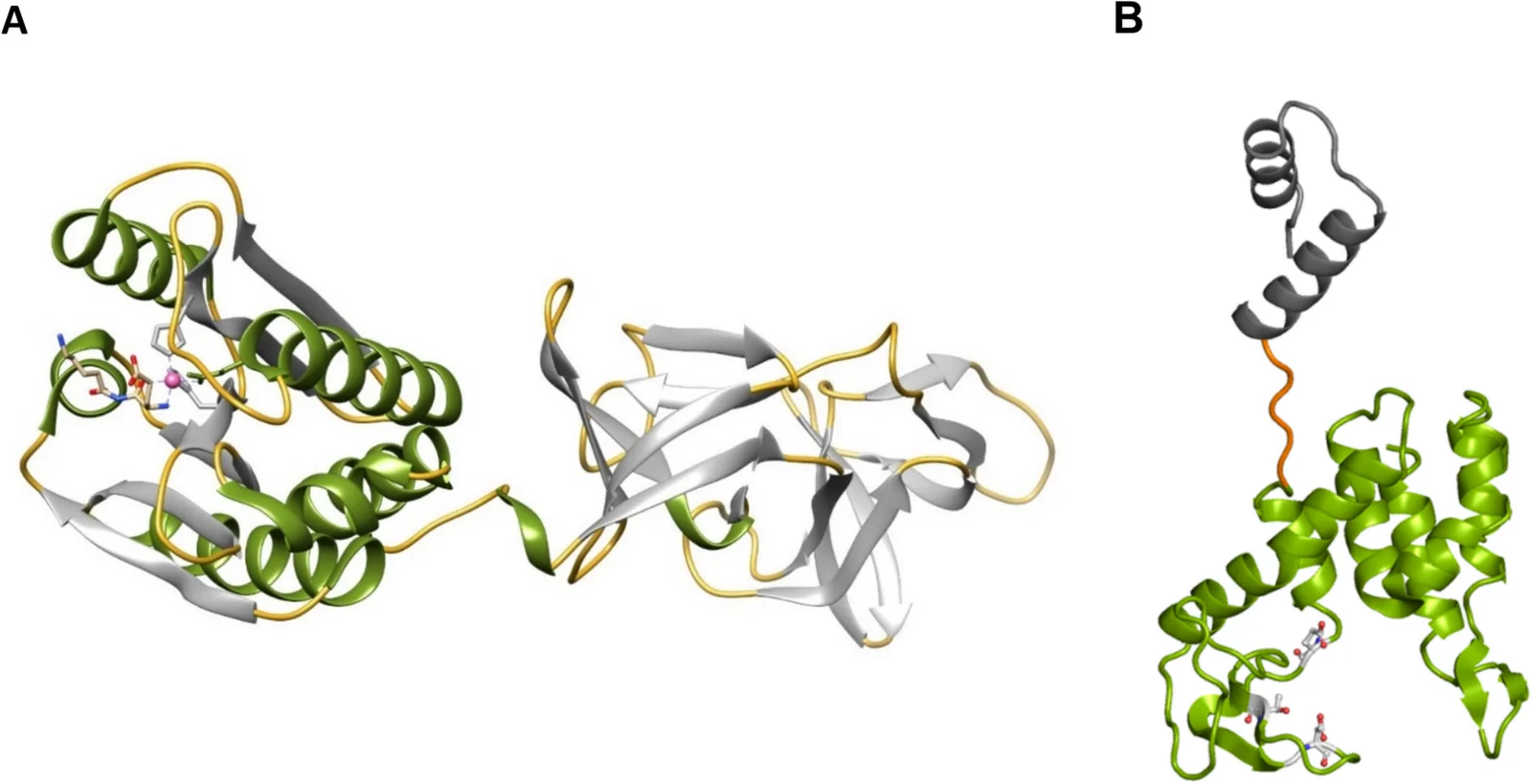
Representative structures of endolysins that target Gram-positive bacteria (**A**) and Gram-negative bacteria (**B**). **A** The crystal structure of the *Listeria monocytogenes* bacteriophage PSA endolysin PlyPSA (PDB code 1XOV) that target Gram-positive bacteria. The architecture of the enzyme with its two separate domains is shown. The two functional domains of the polypeptide, providing cell wall-binding and enzymatic activities, can be clearly distinguished and are connected via a linker segment of six amino acid residues. The core of the N-acetylmuramoyl-L-alanine amidase moiety is formed by a twisted, six-stranded beta-sheet flanked by six helices. Active site residues are depicted as sticks, and the catalytic Zn^2^⁺ ion is represented as a magenta sphere. The C-terminal domain comprising two copies of a beta-barrel-like motif. **B** The structure of endolysin *Ab*Lys1 from *Acinetobacter baumannii* phage AbTZA1, targeting Gram-negative bacteria (PDB code 8 APP, Premetis et al. 2023a). The structure of *Ab*Lys1 consists of two helical domains: a smaller, antenna-like N-terminal domain (color grey) composed of two α-helices and a larger C-terminal domain (colored olive) featuring six α-helices and short β-strands. The smaller domain provides antimicrobial function and the large the catalytic function. Active site residues are depicted as sticks. The two domains are connected via a linker segment (colored orange). The figure illustrates selected representative examples and does not reflect the complete structural diversity observed among endolysins

Endolysins are classified mainly based on three criteria: (i) enzymatic activity/catalytic mechanisms, (ii) domain architecture, and (iii) target specificity. Table 1 summarizes the classification of endolysins based on these three criteria. The structure of endolysins exhibits considerable diversity, with variations in both their modular organization and functional domains (Vázquez et al. 2021; Premetis et al. 2023a, b). This structural variability allows endolysins to target a wide range of bacterial species and adapt to different environmental conditions. The structure of endolysins (Fig. 1) that target Gram-positive bacteria are composed by two distinct structural/functional units. The cell wall binding domain (CBD) and the catalytic (hydrolase) domain (CD). These are linked by flexible connecting regions (Vázquez et al. 2021; Premetis et al. 2023c; Varotsou et al.^2023^; Behera et al. 2024; Khan et al. 2024b). The N-terminal structural unit of endolysins contains the CD with the active site that performs the hydrolysis of peptidoglycan, while the C-terminal structural unit contains the CBD, responsible for the recognition and attachment to the bacterial cell wall. Endolysins that target Gram-negative bacteria are typically small spherical proteins composed of a single functional domain (Vázquez et al. 2021; Premetis et al. 2023a). Some phage-encoded endolysins possess signal-anchor-release (SAR) domains at their N-terminus (Gontijo et al. 2022; Xu et al. 2004). SAR domains are transmembrane regions characterized by a high content of glycine and alanine and a low content of basic amino acids. SAR endolysins hold significant promise as antimicrobial proteins that can act externally on Gram-negative bacteria. These later endolysins possess regions (mainly at N-terminus) with features similar to cationic peptides, enabling them to traverse the outer membrane (Varotsou et al. 2023). These segments enhance the endolysin’s affinity for the bacterial surface and facilitate membrane permeabilization. This strategy is employed for engineering artificial endolysins (Artilysins) and involves the fusion of endolysin molecule with positively charged antimicrobial peptide specifically designed for outer membrane permeation, addressing current challenges in combating Gram-negative bacteria (Briers et al. 2014; Carratalá et al. 2023; Varotsou et al. 2023; Sui et al. 2023). Numerous studies have demonstrated their synergistic activity when combined with antibiotics and antimicrobial peptides (Behera et al. 2024; Lu et al. 2023; Tyagi et al. 2024). Other outer membrane permeabilizers, such as organic acids, EDTA, polymyxins, silver nanoparticles, or liposomes enhance the activity of endolysins synergistically, although certain endolysins are capable of permeating the outer membrane of Gram-negative bacteria independently (Briers et al. 2014; Carratalá et al. 2023; Varotsou et al. 2023; Sui et al. 2023; Behera et al. 2024; Lu et al. 2023; Tyagi et al. 2024).

**Table 1:** Classification of endolysins based on various criteria, such as their enzymatic activity/catalytic mechanisms, domain architecture, and target specificity

Criterion	Categories
Enzymatic activity	Glycosidases (glycan-hydrolyzing enzymes): Muramidases (Lysozymes): Cleave the β−1,4 linkage between N-acetylmuramic acid (MurNAc) and N-acetylglucosamine (GlcNAc) Glucosaminidases: Cleave the β−1,4 linkage between N-acetylglucosamine (GlcNAc) and N-acetylmuramic acid (MurNAc) Amidases (amidohydrolases): Hydrolyze the amide bond between the MurNAc moiety and the stem peptide Endopeptidases: Target specific peptide bonds within the stem peptide or cross-linking peptides Lytic transglycosylases: Cleave β−1,4 glycosidic bonds like muramidases but produce a 1,6-anhydro ring on the MurNAc residue
Domain architecture	Endolysins typically have a modular structure with distinct domains. They can be monomodular (a single catalytic domain) or multimodular (contain both catalytic and cell wall-binding domain) Catalytic domain (CD): Responsible for enzymatic cleavage of the peptidoglycan. Located at either the N-terminus or C-terminus Cell wall-binding domain (CBD): Mediates binding to specific components of the bacterial cell wall, ensuring specificity. Found in most endolysins, often at the opposite terminus to the catalytic domain
Target specificity	Gram-positive specific endolysins: More straightforward access to the peptidoglycan due to the absence of an outer membrane. Often have a CBD for selective targeting Gram-negative specific endolysins: Must traverse or bypass the outer membrane to reach the peptidoglycan. Typically lack a CBD and rely on additional mechanisms (e.g., outer membrane permeabilizers) Broad-spectrum endolysins: Target multiple bacterial species, often by cleaving conserved peptidoglycan components Narrow-spectrum endolysins: Exhibit high specificity for certain bacterial strains, often due to the CBD

The urgent need for innovative solutions to combat bacterial multidrug resistance highlights endolysins as promising candidates for therapeutic applications against both Gram-positive and Gram-negative bacteria. However, several challenges remain to be addressed, including enhancing enzymatic activity, specificity, reducing production costs, improving stability, and optimizing delivery mechanisms to target sites (Antonova et al. 2024; Park et al. 2024; Blanco et al. 2024). Overcoming these key research challenges will be critical to establishing endolysins as clinically validated biological drugs for addressing the global multidrug resistance crisis (Gontijo et al. 2021; Danis-Wlodarczyk et al. 2021). Interestingly, Jansson et al. (2024) have recently shown that synthetic mRNA applied to three human cell lines led to the expression and cytosolic accumulation of the Cpl-1 endolysin with activity against *Streptococcus pneumoniae*. The approach showed potential for the effective treatment against pneumococcal disease.

## Mining endolysin sequences through metagenomics

**Fig. 2:**
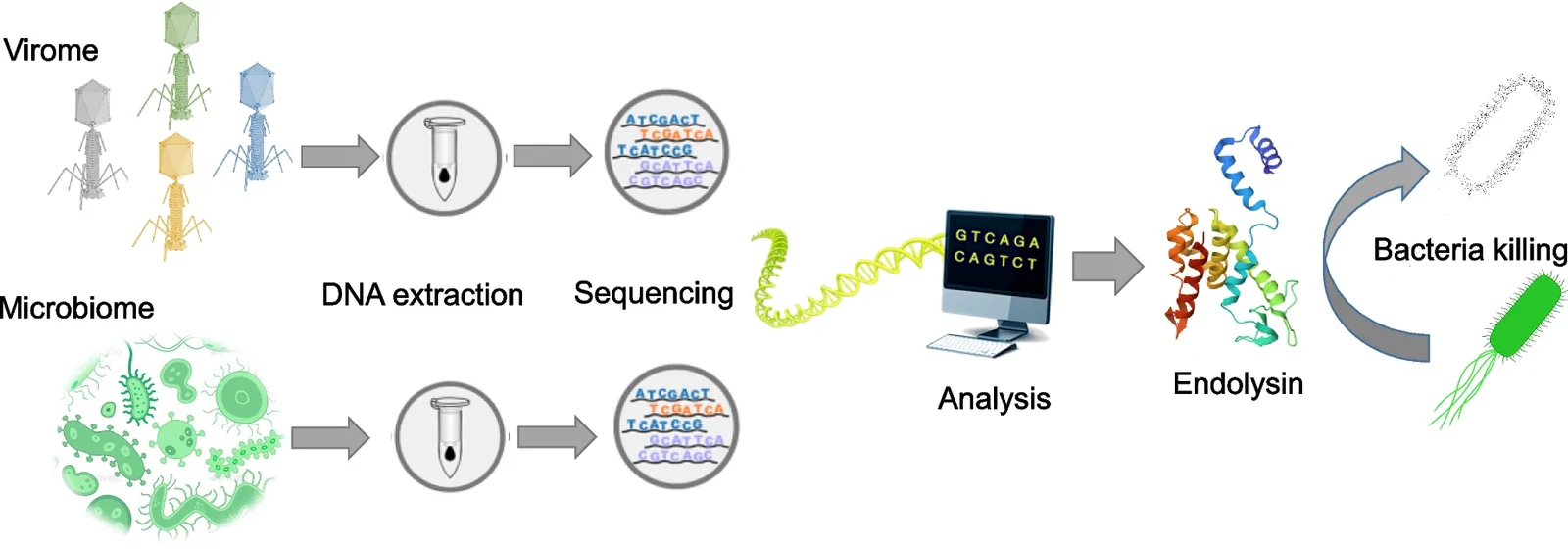
The metagenomics pipeline for the identification of endolysins

Metagenomics allows the study of genetic resources from total genomic DNA of an environmental sample (Robinson et al. 2021; Hogg et al. 2024) (Fig. 2). The sequencing of DNA from microbial communities unlocks access to a vast and diverse reservoir of genetic resources, offering the potential to uncover previously uncharacterized enzymes (Pantiora et al. 2024; Premetis et al. 2023a). It offers a significant advantage over traditional methods, which often depend on isolating and culturing microorganisms that might not represent the full microbial diversity in an environment.

Metagenomics can generate vast and diverse genetic information not only from uncultured microorganisms, but also from uncultured bacteriophages, and the obtained massive data can be exploited for expanding our knowledge on endolysin diversity and evolution (Beliaeva et al. 2023; Oechslin et al 2024; Premetis et al. 2023a). The approach has revolutionized and transformed the search for identifying novel endolysin sequences with desirable traits or improved properties, such as thermostability, specificity, or resistance to environmental degradation, which are crucial for therapeutic and industrial applications (Pantiora et al. 2024; Doss et al. 2023; Oechslin et al. 2024). The high diversity in structure and function of endolysins is partially attributed to their ability to adapt to their new phage/host environment by acquiring adaptive mutations, highlighting the dynamic ability of phage lytic systems to adapt and evolve (Oechslin et al. 2022). The extensive degree of evolvability of endolysins offers new perspectives for their natural engineering and design approaches that can be explored by metagenomics analysis. In addition, metagenomics can shed light on the complexity and diversity of microbial genetic resources, offering insights on the biodiversity-shaping factors that affect the bacteriophage–endolysin–bacteria ecology.

The extensive number of endolysins that can be predicted through metagenomics, presents substantial challenges in selecting high-confidence endolysin sequences for experimental validation. For example, (Fernández-Ruiz et al. 2018) demonstrated the effectiveness of metagenomics for the identification of endolysins from unculturable viral genomes. They analyzed approximately 200,000 uncultured bacteriophage genomes, identifying 2628 putative endolysins, including endolysins with novel domain architectures. This work provided extensive genetic data on endolysin, enhancing our understanding of endolysin diversity and evolution. In another work, (Gontijo et al. 2021) have identified 53 new SAR endolysins among 2628 endolysin genes from 183,298 phage genomes, allowing the enrichment of potential endolysins targeting Gram-negative bacteria.

## Summary of methods for analyzing a microbial community

Advances in DNA sequencing technologies and associated data analysis techniques have significantly improved our ability to study microbial diversity. These developments enable more accurate taxonomic classification of detected organisms while minimizing errors associated with earlier methods (Knight et al. 2018). The massive volume of short reads in metagenomic datasets poses significant computational challenges for genome assembly, function annotation, and meaningful sequence identification. Depending on the experimental objectives, various approaches are available for evaluating microbial communities in environmental samples, including marker gene analysis (Knight et al. 2018), shotgun metagenomic sequencing (Quince et al. 2017), and metatranscriptomic sequencing (Shakya et al. 2019). Each of these methods offers distinct insights, advantages, and limitations (Knight et al. 2018). Among them, shotgun metagenomics offers unexploited potential for discovering novel enzymes directly from environmental DNA, bypassing the typical biases associated with PCR-based or activity-driven functional metagenomics approaches (Robinson et al. 2021; Sime et al. 2024; Premetis et al. 2023a). This approach provides detailed information on the taxonomic diversity, functional potential, and genomic content of microorganisms, including the identification of novel functional enzymes. It also enables the recovery of complete genomes and can also be used for the analysis of viral and eukaryotic DNA within a sample (Premetis et al. 2023a; Quince et al. 2017).

## Sequence-based and functional screening for the identification of endolysins

Sequence-based and functional screening are complementary approaches, each with its advantages and limitations, which can be used for addressing the screening challenges. Sequence-based methods allow rapid exploration of novel enzymes, although they are hindered by the fact that over 30% of prokaryotic genes are annotated as hypothetical, with either general function predictions or without any description due to low homology with known genes. Many sequences represent unknown or uncharacterized proteins, requiring advanced bioinformatics tools, artificial intelligence, and machine learning to predict functions and prioritize candidates for experimental validation (Bałdysz et al. 2024a; Fu et al. 2024). Therefore, the predicted sequences should be ranked based on multiple criteria, such as predicted structure confidence, active site architecture, solubility, and activity, facilitating the selection of high-confidence sequences for experimental validation (Markus et al. 2023). Current databases dedicated to endolysins, such as EnzyBase, phiBIOTICS, PhaLP, and LEDGOs, (Wu et al. 2012; Hojckova et al. 2013; Criel et al. 2021; Mitchell et al. 2021) face several limitations. Most are relatively small in scope, often containing fewer than 1000 entries, and rely primarily on in silico predictions rather than experimentally validated data (Bałdysz et al. 2024a). These limitations reduce their utility for broader comparative studies and for identifying novel endolysins with diverse functionalities.

Bałdysz et al. (2024b) recently provided a critical evaluation of bioinformatic resources available to lysin researchers. They assessed various tools and databases—particularly those based on Hidden Markov Models (HMMs)—commonly used for endolysin analysis. Importantly, the authors introduced a newly curated and comprehensive set of endolysin-related domain and family models, organized into clusters representing major endolysin families, and demonstrated their effectiveness in identifying novel endolysins.

In addition to traditional sequence-based database searches, new algorithms and strategies, such as EnzymeMiner (Hon et al. 2020), FoldSeek (Heinzinger et al. 2024), DeepMineLys (Fu et al. 2024), enable the rapid, and more accurate structure-based searches in metagenomics databases. For example, Fu et al. (2024) have developed DeepMineLys, a convolutional neural network-based framework for identifying phage endolysins from shotgun human microbiome datasets. The strategy allowed the accurate prediction of a large number of endolysin genes. Interestingly, DeepMineLys enabled the identification of an endolysin with 6.2-fold higher activity compared to hen egg white lysozyme.

The resulting sequences can be further optimized through in vitro mutagenesis (Wang et al. 2024a) or in silico machine learning-based tools, such as FuncLib (Khersonsky et al. 2018), MutCompute (Shroff et al. 2020), and Rosetta Cartesian_ddg (Park et al. 2016). Combined with next-generation sequencing, such approaches can produce comprehensive sequence-function datasets. These datasets can facilitate the development of machine learning models, leveraging the extensive range of algorithms currently available (e.g., Feehan et al. 2021; Wang et al. 2024b).

Functional metagenomics, based on enzymatic assays, can address the limitations of sequence-based metagenomics, particularly in screening for novel enzymes (van der Helm et al. 2018). However, the number of highly specialized enzymatic assays available in the literature is limited, and only a small fraction can be directly applied to screening metagenomics libraries (Ngara and Zhang 2018). Early work reported by Schmitz et al. (2008, 2010) established a functional screening protocols for endolysin isolation from genomic (Schmitz et al. 2008) or metagenomic DNA libraries (Schmitz et al. 2010). In the genomic protocol, the procedure begins with mechanical shearing of phage or bacterial DNA into random fragments, followed by end-repair and ligation into plasmid vectors. These recombinant plasmids are then transformed into *Escherichia coli*, allowing the creation of diverse expression libraries in a single day. For functional screening, *E. coli* clones were first permeabilized with chloroform and subsequently overlaid with soft agar containing the target strain (*Bacillus anthracis* ΔSterne in log-phase growth). Clones expressing active endolysins were identified by the absence of bacterial growth around the colonies, indicating peptidoglycan degradation. In the metagenomic protocol (Schmitz et al. 2010), the method was based on a two-step procedure designed to identify endolysins towards *Pseudomonas aeruginosa*. In the first step, viral metagenomic DNA is cloned into plasmids and transformed into *Escherichia coli*. The transformed colonies are then plated on blood agar and exposed to an inducing agent. Clones that exhibit colony lysis and a surrounding hemolytic effect are selected, as this indicates potential expression of phage-derived holins, membrane-permeabilizing proteins that can disrupt the host cell membrane. In the second step, the positive *E. coli* clones identified in the primary step are overlaid with autoclaved *Pseudomonas aeruginosa* cells. This step directly tests for the recombinant expression of phage lytic enzymes, which are often encoded in close proximity to holin genes in phage genomes. The appearance of clear zones in the overlay indicates peptidoglycan degradation and confirms endolysin activity. As a proof of concept, the method was applied to an uncultured viral metagenomic library constructed from mixed animal feces, resulting in the successful cloning of 26 actively expressed lytic enzymes.

## Suitable ecosystems as a source for endolysin discovery through metagenomics

The first step in endolysin discovery through metagenomics is the selection of an appropriate environmental sample. The source of the sample can vary depending on the intended application of the endolysin. For example, if the goal is the isolation of a thermostable endolysin, a hot spring or geothermal environment would be an ideal source (Pantiora et al. 2024). Similarly, for enzymes that might target specific pathogens, environments such as wastewater (Premetis et al. 2023a) or the human microbiome could provide valuable candidates (Fu et al. 2024). In the subsequent sections, we will examine examples of endolysins identified through metagenomic analyses of human microbiome, microbial biofilms, and hot springs/geothermal environments, as these sources encompass diverse ecosystems with significant endolysin diversity (Table 2).

**Table 2:** Overview of characterized endolysin discovery via metagenomics

Source ecosystem	Endolysin name	Approach	Results/target	Reference
Human microbiome	CD27L_EAD	Metagenomic screening of human fecal microbiome	Effective against *C. difficile*; selective without affecting gut microbiota	Cho et al. (2024)
Human microbiome	PolaR	Computational analysis of gastric mucosa phageome	Targets Rothia spp.; active against biofilms; non-cytotoxic to mammalian cells	Miernikiewicz et al. (2023)
Human microbiome	10 endolysins	Phage-host association analysis in human intestinal microbiome	Endolysins against *C. difficile* confirmed in vitro and in vivo	Fujimoto et al. (2020)
Marine biofilm near industrial zone	AbLys2	Viral metagenomics analysis of marine biofilm	Glycoside hydrolase family 24; lytic activity against *Acinetobacter baumannii*	Premetis et al. (2023a)
Octopus Spring biofilm	-	Viral metagenomics and custom annotation pipeline	Endolysins with Glycoside hydrolase family 108 domain	Davison et al. (2016)
Western Mediterranean marine sample	-	PacBio sequencing of marine virome	Recovered > 30,000 unique sequences; several predicted endolysins	Zaragoza-Solas et al. (2022)
Hot spring soil	Ami1	Metagenomic mining of hot spring soil	Broad activity; thermostable (T_m_ 64.2 °C); strong activity against Staphylococcus species	Pantiora et al. (2024)
Thermophilic phages from hot springs	PhiKo endolysin	Characterization of phage infecting *Thermus thermophilus*	Broad activity; thermostable (T_m_ 91.7 °C)	Szadkowska et al. (2024)
Thermophilic phages (various genera)	Ts2631	Isolation from *Thermus scotoductus* bacteriophage	Broad activity; thermostable (T_m_ 99.8 °C)	Plotka et al. (2019)

### Human microbiome

The human microbiome represents a rich and diverse source of endolysins, as it harbors a wide variety of bacteria with distinct cell wall structures (Fujimoto et al. 2020; Cho et al. 2024). Additionally, the human microbiome offers a unique environment for discovering endolysins with broad-spectrum specificity due to its complexity and dynamic microbial interactions. For example, Cho et al. (2024) have identified through a metagenomics screening of the human fecal microbiome the endolysin CD27L_EAD. This enzyme is effective against *Clostridium difficile*, a major cause of antibiotic-resistant gastrointestinal infections. The enzyme showed bactericidal activity against a broad range of *C. difficile* strains, including both toxigenic and non-toxigenic isolates. Notably, CD27L_EAD did not display activity against beneficial gut microbiota, a significant major advantage for the development of enzyme-based therapeutics for gut infections. Fujimoto et al. (2020) have reported the analysis of intestinal viral and bacterial microbiomes in healthy individuals. Based on the genomic sequences of bacteriomes and viromes, the host bacteria-phage associations were found for both temperate and virulent phages. The authors exploited the host bacteria-phage information, to identify *C. difficile*-specific phages and ten homologous endolysin sequences from the prophage regions in the *C. difficile*. The activity of endolysins was confirmed both in vitro and in vivo.

In another study, Miernikiewicz et al. (2023) have conducted a computational analysis of metagenomic sequencing data from gastric mucosa phageomes. They identified and developed a specific endolysin (PolaR) targeting *Rothia mucilaginosa* and *Rothia dentocariosa*. PolaR was also effective towards bacterial cells in biofilms, without exhibiting cytotoxic or antiproliferative effects on mammalian cells. The authors concluded that since PolaR is the first reported endolysin that specifically targeting *Rothia* species, it holds the potential for treating infections caused by these bacteria and possibly others.

### Microbial biofilms

Microbial biofilms are ecosystems formed in diverse environments such as aquatic environments, waste areas, industrial and hospital settings (Talapko and Škrlec 2020). Regions with high levels of human activity are favorable ecosystems to the formation of biofilms by pathogenic bacteria (e.g., ESKAPE pathogens) (Dunne 2002). Microorganisms within a biofilm exhibit significantly higher resistance to antimicrobial agents, as their gene expression and metabolic pathways differ markedly from those observed during independent growth (Del Pozo 2018; Sharma et al. 2019). For example, Srinivasan et al. (2021) reported that bacteria in biofilm form often require antimicrobial concentrations up to 1,000 times higher than their free-living (planktonic) counterparts to achieve effective inhibition. Therefore, biofilms represent suitable ecosystems for the isolation of novel bacteriophages and endolysins targeting resistant pathogens. As bacteriophages replicate within bacterial cells, their proliferation is remarkably enhanced due to increased number of bacterial cells susceptible to infection in the surrounding environment (Sillankorva et al. 2011). In addition, this localized propagation within the biofilm milieu fosters the continuous interactions between phages and their hosts, enhancing evolutionary adaptations and mutagenesis, which may improve the effectiveness of phages and their endolysins in bacterial lysis (Talapko and Škrlec 2020). In a recent work, reported a metagenomics analysis of the viral diversity of a marine biofilm, formed near an industrial zone. They found wide range of known bacteriophages, which some of them (0.34%) are specific against the human ESKAPE pathogens. The work allowed the mining of a novel endolysin (AbLys2) that belongs to the glycoside hydrolase family 24 and displays lytic activity towards *Acinetobacter baumannii*. In another study, (Davison et al. 2016) conducted viral metagenomics analysis from Octopus Spring biofilms. They developed a custom module that enabled the identification of three phage clusters correlating with host range and predicted 52,348 ORFs, including endolysin sequences. Analysis of the endolysin sequences within the thermophilic cyanophage contigs, allowed the finding of a group of well-characterized endolysins alongside the Glyco_hydro_108 (PF05838) domain. Notably, a Glyco_hydro_108 domain, previously unlinked to cyanophages, was identified. Zaragoza-Solas et al. (2022) compared viral diversity in a marine sample from the western Mediterranean. Using PacBio circular consensus sequencing, they achieved the recovery of over 30,000 unique sequences, many without homologues in assemblies or the Global Ocean Virome database (Wu et al. 2023) and predicted as endolysins.

### Hot springs and geothermal areas are potential sources of thermostable endolysins

Thermostable enzymes are receiving considerable attention and there are already many industrial applications (Jaiswal and Jaiswal 2024). The interest in thermostable endolysins is derived from their intrinsic properties such as prolonged storage, protease resistance as well as low activity losses during long applications. Therefore, they can be superior tools in medical and technical processes (Pantiora et al. 2024).

Hot springs and geothermal areas are potential sources of diverse arrays of microbes, associated viruses (“thermophilic phages”) and their thermostable endolysins (Zablocki et al. 2018; Szadkowska et al. 2024). The potential for discovery of novel phage-derived endolysins from terrestrial hot springs remains mostly untapped. Recent studies have established that endolysins from thermophilic bacteriophages possess thermostable endolysins that can be used in food processing and in veterinary medicine. For example, the endolysin from the phiKo bacteriophage that infects the Gram-negative *Thermus thermophilus* HB27 and other mesophiles, displays a melting temperature of 91.70 °C (Szadkowska et al. 2024). The endolysin Ts2631 from *Thermus scotoductus* bacteriophage shows high lytic activity not only against thermophiles but also against Gram-negative mesophilic bacteria. It exhibits a melting temperature of 99.8 °C (Plotka et al. 2019). Similarly, Doss et al. (2023) reported that the endolysins from bacteriophages infecting the genera *Thermus*, *Meiothermus* and *Geobacillus* exhibit high stability and unusually broad lytic activity against Gram-negative and Gram-positive bacteria.

The study by Pantiora et al. (2024) focuses on mining the metagenome of hot spring soil samples to identify novel, thermostable endolysins. The identified prophage-derived endolysin, Ami1, revealed that it belongs to the N-acetylmuramoyl-L-alanine amidase type 2 family and contains a LysM cell wall binding domain. Ami1 demonstrated strong bactericidal and antimicrobial activity against a broad range of bacterial pathogens, particularly *Staphylococcus aureus* and *Staphylococcus epidermidis*. Furthermore, the enzyme displays stability at high temperatures and exhibits melting temperature 64.2 ± 0.6 °C.

## Conclusions

Metagenomics provides an exciting and powerful tool for discovering novel enzymes from diverse and underexplored environmental sources. While metagenomics holds great promise for enzyme discovery, it is still constrained by a number of critical challenges. One significant issue is the complexity of large libraries, which, combined with the sequence and structural diversity of endolysins, makes the confident identification of novel endolysin sequences difficult. The identification of active enzymes can be time-consuming and resource-intensive, requiring high-throughput screening technologies and robust functional assays. Looking ahead, improvements in high-throughput sequencing, functional screening methods and bioinformatics tools will help accelerate the discovery of novel endolysins.

As novel endolysins are identified through metagenomic approaches, their clinical potential is increasingly being realized. Several candidates have advanced to preclinical and early clinical stages, showing promising results in terms of safety and efficacy (Antonova et al. 2024; Jansson et al. 2024). However, key challenges remain, including ensuring stability in physiological environments, overcoming immune responses, achieving effective delivery, especially across the outer membrane of Gram-negative bacteria, and scaling up cost-effective production (Ho et al. 2022; Sisson et al. 2024). Ongoing research in protein engineering, such as the development of Artilysins, PEGylated variants, and chimeric domain-fused enzymes, is addressing these hurdles and paving the way for the clinical translation of endolysin-based therapeutics (Briers et al. 2014; Behera et al. 2024; Park et al. 2024).

## Data Availability

Not applicable.
